# Association between neutrophil percentage-to-albumin ratio and mortality in patients with community acquired pneumonia receiving systemic glucocorticoids: a retrospective cohort study

**DOI:** 10.3389/fmed.2025.1689323

**Published:** 2025-10-13

**Authors:** Juan Guo, Chunjing Jiang, Juanyou Ran, Li Tang

**Affiliations:** ^1^Department of Respiratory Medicine, Chongqing Emergency Medical Center, Chongqing University Central Hospital, Chongqing, China; ^2^Department of Neurology, Chongqing Emergency Medical Center, Chongqing University Central Hospital, Chongqing, China; ^3^Department of Central Sterile Supply, Chongqing Emergency Medical Center, Chongqing University Central Hospital, Chongqing, China

**Keywords:** neutrophil percentage-to-albumin ratio, glucocorticoids, mortality, community acquired pneumonia, Dryad database

## Abstract

**Background:**

The ratio of neutrophil percentage to albumin (NPAR) has been recognized as an inflammatory indicator for predicting the prognosis of various diseases. Nevertheless, no research has explored the relationship between NPAR and prognosis in patients who develop community acquired pneumonia (CAP) during long-term and systemic glucocorticoids therapy. Therefore, this study aims to investigate the association between NPAR on admission and mortality in the aforementioned patients.

**Method:**

The data of this study were extracted from the Dryad database. An analysis was conducted data from patients diagnosed with CAP who had received either oral or intravenous glucocorticoids before hospital admission. Patients were categorized into three groups based on their NPAR levels upon admission. Kaplan-Meier survival curves, multivariable Cox regression models, restricted cubic spline curves, and subgroup analyses were performed to evaluate the association between the NPAR and 30-day as well as 90-day mortality in these patients, respectively. Sensitivity analysis were performed to verify the stability of the results.

**Results:**

Among the 570 patients diagnosed with CAP incorporated into the study, the 30-day and 90-day mortality were 21.9% and 24.9%, respectively. The study revealed that the NPAR exhibited a significantly positive correlation with mortality. Multivariable Cox regression analyses, after adjustment for all possible confounders, indicated that a higher NPAR level was correlated with an elevated risk of 30-day mortality (HR: 1.21, 95% CI: 1.14–1.28). Compared with patients in tertile 1, those in tertile 2 and tertile 3 exhibited a notably increased risk of 30-day mortality (HR: 1. 83, 95% CI: 1. 38–2. 43; HR: 3. 19, 95% CI: 2. 72–4. 2, respectively). Analogous findings were also observed for 90-day mortality. Kaplan-Meier survival curves showed that the highest tertile had the lowest survival rates for 30-day and 90-day mortality. Additionally, subgroup analysis revealed no interactions and demonstrated robust results across different subgroups. A linear relationship was observed between NPAR and mortality.

**Conclusion:**

Higher level of NPAR was significantly associated with an increased risk of 30-day and 90-day mortality in patients with community acquired pneumonia receiving systemic glucocorticoids therapy.

## 1 Introduction

Community-acquired pneumonia (CAP) is a common condition that results in high morbidity and mortality worldwide, imposing substantial healthcare burdens (1, 2). Due to underlying conditions including connective tissue diseases (3), chronic lung diseases (4), nephrotic syndrome or chronic glomerulonephritis (5), hematological disorders (6), etc., a subset of patients requires long-term use of systemic glucocorticoid therapy. The risk of pulmonary infections, including CAP, has significantly increased in this population (7–10). Furthermore, studies have also indicated that these patients often exhibit increased disease severity and higher mortality rates upon developing CAP compared to those not using systemic steroids (11). Thus, reliable scoring systems or indicators are needed to guide clinical decisions and evaluate the prognosis of these pneumonia patients.

At present, both the CURB-65 (confusion, urea nitrogen, respiratory rate, blood pressure, age ≥ 65 years) and pneumonia severity index (PSI) serve as assessment tools for the severity of illness and prognosis of patients with CAP in clinical practice (12, 13). Although these scores have certain application value, they have limitations when applied to specific populations. Both prognostic scoring systems are primarily used for immunocompetent populations; however, their prognostic accuracy diminishes in immunosuppressed populations (10, 14), such as glucocorticoid-treated patients who develop CAP. Certain inflammatory biomarkers, including leukocyte count, procalcitonin (PCT), and C-reactive protein (CRP), have also been utilized to evaluate the prognosis of pneumonia (15). Nevertheless, the levels of these biomarkers might be affected by the immunosuppressive effects of glucocorticoids (16). Consequently, it is crucial to explore novel and effective prognostic markers to evaluate the mortality risk in patients with pneumonia receiving glucocorticoids therapy.

The neutrophil percentage-to-albumin ratio (NPAR), a novel inflammatory biomarker, integrates the neutrophil percentage and albumin and exhibits a substantial correlation with inflammatory reaction and nutritional status of the body. Previous researches have indicated that NPAR was significantly associated with the prognosis of various diseases, including those aged 80 years or older with CAP (17), sepsis (18), cardiovascular diseases (19), stroke (20), acute kidney injury (21), etc. Although NPAR has been linked to a certain predictive value for the prognosis of patients with different diseases, its role in predicting mortality among pneumonia patients receiving glucocorticoids remains unclear. Therefore, the present study investigates the correlation between NPAR and mortality among CAP patients treated with long-term and systemic glucocorticoids, aiming to identify potential prognostic indicators to enhance clinical management for this high-risk population.

## 2 Materials and methods

### 2.1 Data source

The data were obtained from the Dryad Digital Repository,^1^ which allows unrestricted use of the data for other researchers. The dataset containing 716 pneumonia patients who received oral or intravenous glucocorticoids treatment was firstly provided by Li et al. (8). These patients were diagnosed with pneumonia upon admission or during hospitalization at six secondary and tertiary academic hospitals in China between January 2013 and December 2017 (8).

### 2.2 Study population

**FIGURE 1:**
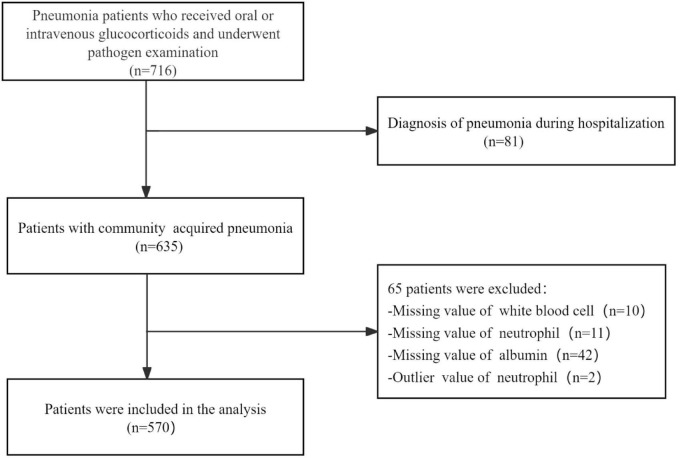
Flow chart of study population.

This study included patients aged 16 years or older who had received oral or intravenous glucocorticoids therapy before admission and were hospitalized due to CAP. The diagnosis of CAP was performed in accordance with the guideline of the American Thoracic Society and Infectious Disease Society of America (22). Exclusion criteria were as follows: (1) inability to provide informed consent; (2) hospital-acquired pneumonia; (3) missing values for white blood cell, neutrophil, and albumin; (4) Outlier values of neutrophil. Ultimately, 570 patients were included in the study for subsequent analysis (Figure 1).

### 2.3 Data extraction

The covariates in this study were selected based on previous research, including: (1) demographic data, such as age and gender; (2) underlying diseases, which comprised connective tissue diseases (CTD), chronic obstructive pulmonary disease (COPD), interstitial lung disease (ILD), hematological disorders, and idiopathic interstitial pneumonia (IIP), nephrotic syndrome or chronic renal failure (CRF), liver failure, cirrhosis, nephrotic syndrome, congestive heart disease, or tumor, etc; (3) pneumonia severity scoring systems, including CURB-65 (confusion, urea nitrogen, respiratory rate, blood pressure, age ≥ 65 years) and the Pneumonia Severity Index (PSI); (4) laboratory data, which included white blood cell (WBC), neutrophil, lymphocyte, hemoglobin, platelet, albumin, lactate dehydrogenase (LDH), blood urea nitrogen (BUN), serum creatinine, procalcitonin (PCT), and the total pathogenic positive rate; (5) therapeutic interventions, such as use of high - dose glucocorticoids, cumulative methylprednisolone dosages, oxygen inhalation, intensive care unit (ICU) admission, mechanical ventilation, intubation, extracorporeal membrane oxygenation (ECMO), continuous veno-venous hemofiltration (CVVH), and vasoactive drugs; (6) survival status at 30 days and 90 days post-admission. All the laboratory tests mentioned above were collected within 24 h of admission.

The neutrophil percentage was delineated as the proportion of neutrophil in the total white blood cell count. The NPAR was calculated via the formula: (Neutrophil percentage × 100)/Albumin (g/dL), utilizing the identical blood samples collected upon admission. High-dose glucocorticoids was defined as the administration of 30 mg/day or more of prednisolone, or an equivalent glucocorticoids within the 30 days preceding admission (23). Persistent lymphocytopenia was characterized as a peripheral blood lymphocyte count below 1 × 10^9^/L persisting for more than 7 days (24).

### 2.4 Statistical analysis

Normally distributed continuous variables were expressed as mean ± standard deviation (SD), and skewedly distributed continuous variables were described using median with interquartile range (IQR). While categorical variables were delineated by frequencies with percentages. To eliminate the dimensional differences of NPAR, it was z-score transformed before analysis.

Upon reviewing previous literature, patients were categorized into tertiles according to NPAR levels upon admission [tertile 1 (T1): NPAR < 0.218; tertile 2 (T2): NPAR ≥ 0.218, <0.275; tertile 3 (T3): NPAR ≥ 0.275], and T1 group served as the reference group. Trend tests across tertiles were conducted using median values. Comparisons between the three groups were conducted using ANOVA test or Kruskal-Wallis for continuous variables, and χχ^2^ test for categorical variables. Variables with a missing rate exceeding 25% were excluded from this analysis. Multiple imputation with five replications was utilized to tackle other missing data. Survival rates between groups were estimated by Kaplan-Meier curves and compared by log-rank test. Univariate Cox regression analyses were utilized to assess the correlation between prognostic factors and 30-day and 90-day mortality. Multivariate analyses were conducted via Cox proportional hazards models to evaluate the independent correlation between the NPAR and mortality. Variables were incorporated into the multivariate Cox proportional hazard models in accordance with clinical experience, the results of univariate regression and an alteration in their effective estimate exceeding 10%. Three models were constructed to control for confounding factors. Model I was adjusted for age and gender. Model II was based on Model I, incorporating the CURB-65, PSI, COPD, and persistent lymphocytopenia. Model III was based on Model II, with the addition of intubation, ICU admission, ventilation, and vasoactive drugs. The outcomes were presented in the form of hazard ratios (HR) accompanied by 95% confidence intervals (CI). Restricted cubic splines were used to observe and analyze the relationship between NPAR and mortality. Additionally, subgroup analyses and interactions were conducted using Cox regression models based on age, gender, COPD, CURB-65, persistent lymphocytopenia, mechanical ventilation, and vasoactive drugs. The results were visualized through forest plots. Furthermore, since chronic renal failure, liver failure, cirrhosis, nephrotic syndrome, congestive heart disease, and tumor may affect albumin levels, we excluded these subset of patients and performed sensitivity analyses.

Statistical analyses were carried out using R software (The R Foundation)^2^ and Free Statistics software version 2.2. All tests were two-tailed, and *P*-values < 0.05 were considered statistically significant.

## 3 Results

### 3.1 Baseline characteristics of patients

A total of 570 participants, who received glucocorticoids therapy and subsequently developed community acquired pneumonia, were ultimately included in this study (Figure 1). The baseline characteristics of the pneumonia patients stratified according to tertiles of NPAR, are summarized in Table 1. Each group included 190 patients. Among the study population, 298 (52.3%) were aged 60 years or older, and 265 (46.5%) were female. The most prevalent underlying diseases among those receiving glucocorticoid therapy were CTD (52.8%), ILD (45.8%), and COPD (16.8%). Patients in the highest NPAR tertile exhibited higher CURB-65 and PSI scores, and may need to be admitted to the ICU for treatment compared to those in the other tertiles. As NPAR increased, WBC, neutrophils, LDH, BUN, creatinine, and procalcitonin levels increased, whereas their oxygenation index, hemoglobin and albumin levels decreased. Furthermore, patients with a higher NPAR also experienced persistent lymphocytopenia, along with increased use of oxygen inhalation, intubation, ventilators, vasoactive drugs, and CVVH compared to those in the other tertiles. The 30-day and 90-day mortality rates were documented as 21.9% and 24.9%, respectively.

**TABLE 1:** Baseline characteristics of the study population by NPAR tertiles.

Characteristics	Total (*n* = 570)	NPAR			*P*-value
		<0.218 (*n* = 190)	≥0.218, <0.275 (*n* = 190)	≥0.275 (*n* = 190)	
Age ≥ 60 years, *n* (%)	298 (52.3)	95 (50.0)	97 (51.1)	106 (55.8)	0.485
Female, *n* (%)	265 (46.5)	94 (49.5)	85 (44.7)	86 (45.3)	0.598
**Underlying diseases, *n* (%)**					
COPD	96 (16.8)	37 (19.5)	31 (16.3)	28 (14.7)	0.454
ILD	261 (45.8)	91 (47.9)	97 (51.1)	73 (38.4)	0.037
Bronchial asthma	15 (2.6)	8 (4.2)	4 (2.1)	3 (1.6)	0.237
CTD	301 (52.8)	100 (52.6)	103 (54.2)	98 (51.6)	0.875
IIP	64 (11.2)	21 (11.1)	30 (15.8)	13 (6.8)	0.022
Hematonosis	63 (11.1)	20 (10.5)	19 (10.0)	24 (12.6)	0.687
Nephrotic syndrome or CRF	88 (15.4)	16 (8.4)	28 (14.7)	44 (23.2)	<0.001
**Scoring systems**					
CURB-65 > 1, *n* (%)	160 (28.1)	35 (18.4)	53 (27.9)	72 (37.9)	<0.001
PSI, mean ± SD	80.0 ± 31.1	68.6 ± 25.5	81.5 ± 31.8	89.9 ± 31.9	<0.001
**Laboratory parameters**					
WBC (×10^9^/L), mean ± SD	9.2 ± 5.6	8.0 ± 6.4	9.5 ± 4.8	10.2 ± 5.2	<0.001
Neutrophil (×10^9^/L), mean ± SD	7.5 ± 4.9	5.5 ± 4.8	7.9 ± 4.3	9.2 ± 4.8	<0.001
Lymphocyte (×10^9^/L), median (IQR)	0.8 (0.5, 1.4)	1.3 (0.9, 1.9)	0.9 (0.6, 1.2)	0.5 (0.3, 0.8)	<0.001
Hemoglobin (g/L), mean ± SD	113.2 ± 22.9	118.5 ± 24.0	113.8 ± 20.5	107.3 ± 22.7	<0.001
Platelet (×10^9^/L), mean ± SD	190.9 ± 88.8	199.2 ± 82.7	190.3 ± 77.7	183.0 ± 103.7	0.212
Albumin (g/dL), mean ± SD	3.3 ± 0.6	3.8 ± 0.5	3.3 ± 0.3	2.7 ± 0.4	<0.001
AST (U/L), median (IQR)	23.0 (16.0, 38.0)	19.0 (15.0, 26.0)	25.0 (17.0, 39.3)	30.0 (17.5, 46.0)	<0.001
ALT (U/L), median (IQR)	24.0 (15.0, 45.0)	19.5 (14.0, 33.0)	27.0 (17.0, 53.0)	26.0 (16.0, 48.0)	<0.001
BUN (mmol/L), median (IQR)	6.2 (4.6, 9.5)	5.2 (3.9, 7.9)	6.2 (4.7, 9.5)	7.4 (5.5, 11.4)	<0.001
Serum creatinine (mmol/L), median (IQR)	64.2 (51.5, 89.0)	62.9 (52.8, 78.6)	61.9 (49.0, 83.3)	73.5 (52.2, 107.3)	0.01
LDH (U/L), median (IQR)	313.5 (225.0, 478.8)	237.0 (188.4, 328.2)	327.5 (254.5, 469.2)	421.5 (270.5, 576.0)	<0.001
ESR (mm/h), median (IQR)	38.0 (18.0, 68.0)	32.0 (12.8, 66.2)	38.0 (16.0, 62.0)	48.0 (29.5, 73.0)	<0.001
Procalcitonin (ng/mL), median (IQR)	0.3 (0.1, 0.8)	0.2 (0.1, 0.4)	0.3 (0.2, 0.7)	0.4 (0.2, 1.3)	<0.001
Oxygenation index, mean ± SD	254.6 ± 138.5	317.8 ± 121.8	261.2 ± 133.5	198.3 ± 133.2	<0.001
Persistent lymphocytopenia, *n* (%)	239 (41.9)	43 (22.6)	80 (42.1)	116 (61.1)	<0.001
Total pathogenic positive rate, *n* (%)	405 (87.9)	123 (84.8)	139 (89.1)	143 (89.4)	0.403
**Treatment, *n* (%)**					
Cumulative methylprednisolone dosages	4.0 (2.2, 8.8)	4.5 (2.1, 12.1)	4.1 (2.2, 7.9)	3.6 (2.2, 6.7)	0.289
High-dose glucocorticoids	202 (35.4)	38 (20.0)	72 (37.9)	92 (48.4)	<0.001
**Complications, *n* (%)**					
Oxygeninhalation	409 (72.0)	108 (56.8)	139 (73.2)	162 (86.2)	<0.001
ICU admission	226 (39.6)	39 (20.5)	74 (38.9)	113 (59.5)	<0.001
Intubation	118 (24.8)	21 (13.2)	39 (24.4)	58 (36.9)	<0.001
Mechanical ventilation	194 (34.0)	32 (16.8)	63 (33.2)	99 (52.1)	<0.001
ECMO	29 (5.1)	8 (4.2)	10 (5.3)	11 (5.80)	0.775
Vasoactive drugs	98 (17.2)	15 (7.9)	34 (17.9)	49 (25.9)	<0.001
CVVH	51 (8.9)	9 (4.7)	15 (7.9)	27 (14.2)	0.004
**Outcomes, *n* (%)**					
30-day mortality	125 (21.9)	15 (7.9)	35 (18.4)	75 (39.5)	<0.001
90-day mortality	142 (24.9)	19 (10.0)	42 (22.1)	81 (42.6)	<0.001

Data are presented as *n* (%), mean ± SD, and median (IQR). NPAR, neutrophil percentage-to-albumin ratio; COPD, chronic obstructive pulmonary disease; ILD, interstitial lung diseases; CTD, connective tissue disease; IIP, idiopathic interstitial pneumonia; Hematonosis, anemia, leukemia, lymphoma, bonemarrow transplantation; CRF, chronic renal failure; CURB-65, confusion, urea nitrogen, respiratory rate, blood pressure, age ≥ 65 years; PSI, pneumonia severity index; WBC, white blood cell; LDH, lactate dehydrogenase; BUN, blood urea nitrogen; ESR, erythrocyte sedimentation rate; ICU, intensive care unit; ECMO, extracorporeal membrane oxygenation; CVVH, continuous venovenous hemofiltration.

### 3.2 Kaplan–Meier curves

**FIGURE 2:**
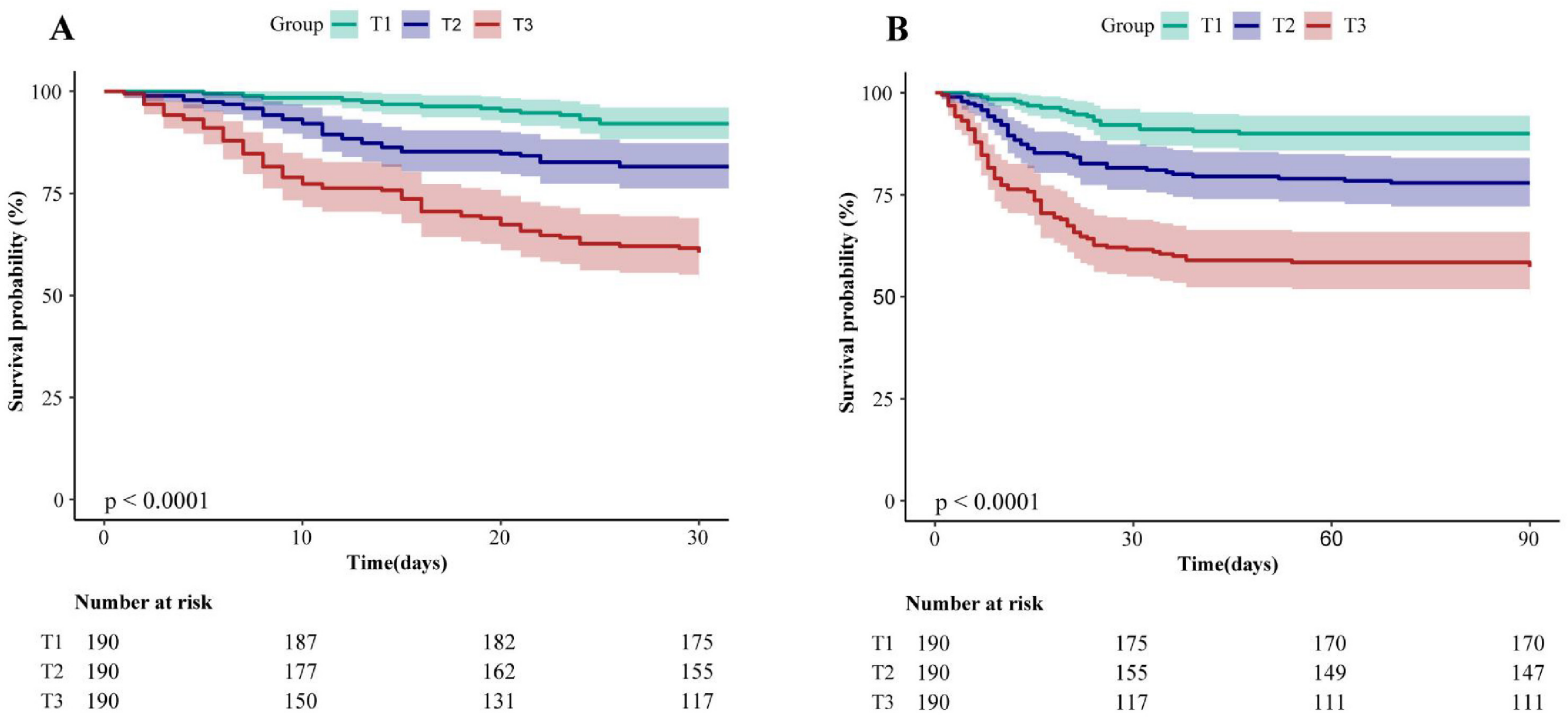
Kaplan–Meier analysis of 30-day **(A)** and 90-day **(B)** mortality for patients with CAP receiving glucocorticoids therapy (log-rank test, *P* < 0.0001). NPAR, neutrophil percentage-to-albumin ratio; T, Tertile; T1: <0.218, T2: ≥0.218, <0.275, T3: ≥0.275.

The Kaplan-Meier survival curves illustrating the 30-day and 90-day mortality rates classified by the NPAR tertiles were presented in Figure 2. A notable discrepancy was observed among each group. Throughout the follow-up period, the 30-day survival rate in group T3 was significantly lower than those in groups T1 and T2 (log-rank test: *P* < 0.0001) (Figure 2A). Similar trends were found in 90-day survival rate (log-rank test: *P* < 0.0001) (Figure 2B).

### 3.3 Multivariable COX regression analysis

To demonstrate whether the NPAR admission level was associated with 30-day and 90-day mortality in patients with CAP receiving systemic glucocorticoids therapy, univariate Cox regression analyses were conducted and are detailed in Supplementary Table 1. A positive correlation was observed between the NPAR (z-score) and mortality (*P* < 0.001). Subsequently, we further employed three multivariate Cox regression models to evaluate the independent effect of NPAR on 30-day and 90-day mortality (Table 2). When evaluated as a continuous variable, we found that each SD increment of NPAR in unadjusted model, the risk of 30-day mortality was increased by 47% (HR: 1.47; 95% CI: 1.4–1.54). Even after adjusting for all potential confounders in model III, this association still persisted strongly, and the risk of 30-day mortality elevated by 21% (HR: 1.21, 95% CI: 1.14–1.28, *P* < 0.001). To ensure the robustness of the results, we also analyzed the NPAR as a categorical variable according to its tertiles, using the tertile 1 group (T1) as a reference. For the outcome of 30-day mortality, we found that higher NPAR was related to increased risk of mortality. The HRs (95% CI) values of the middle-tertile (T2) and the highest tertile (T3) were 2.52 (1.92–3.3) and 6.26 (4.88–8.02), respectively, when compared with the reference group (T1). Trend testing confirmed a significant association between increasing NPAR levels and mortality (*P* < 0.001). This association remained statistically even after adjusted for relevant covariates. After adjustment for age, gender in model I, an increasing trend was also observed in the highest NPAR tertile (HR: 6.16, 95% CI: 4.81–7.9, *P* < 0.001). After further adjustment for potential confounders (age, gender, CURB-65, PSI, COPD, persistent lymphocytopenia) in model II, the upward trend remained statistically significant in the high tertile (HR: 3.52, 95% CI: 2.72–4.57, *P* < 0.001). In model III, the fully adjusted HRs (95% CI) were 1.83 (95% CI: 1.38–2.43) and 3.19 (95% CI: 2.42–4.2) for tertile 2 and tertile 3, respectively. Furthermore, similar results were also observed for the association between NPAR and 90-day mortality (Table 2).

**TABLE 2:** Multivariable Cox regression to assess the association of NPAR with 30-day and 90-day mortality.

Variable	Non-adjusted Model		Model I		Model II		Model III	
	HR (95% CI)	*P*-value	HR (95% CI)	*P*-value	HR (95% CI)	*P*-value	HR (95% CI)	*P*-value
**30-day mortality**								
NPAR (per SD increment)	1.47 (1.4∼1.54)	<0.001	1.47 (1.4∼1.54)	<0.001	1.28 (1.21∼1.36)	<0.001	1.21 (1.14∼1.28)	<0.001
**NPAR tertile**								
T1 (<0.218)	1 (Ref)		1 (Ref)		1 (Ref)		1 (Ref)	
T2 (≥0.218, <0.275)	2.52 (1.92∼3.3)	<0.001	2.50 (1.91∼3.28)	<0.001	1.75 (1.32∼2.3)	<0.001	1.83 (1.38∼2.43)	<0.001
T3 (≥0.275)	6.26 (4.88∼8.02)	<0.001	6.16 (4.81∼7.9)	<0.001	3.52 (2.72∼4.57)	<0.001	3.19 (2.42∼4.2)	<0.001
*P* for trend		<0.001		<0.001		<0.001		<0.001
**90-day mortality**								
NPAR (per SD increment)	1.46 (1.4∼1.52)	<0.001	1.45 (1.39∼1.52)	<0.001	1.28 (1.21∼1.36)	<0.001	1.21 (1.14∼1.28)	<0.001
**NPAR tertile**								
T1 (<0.218)	1 (Ref)		1 (Ref)		1 (Ref)		1 (Ref)	
T2 (≥0.218, <0.275)	2.40 (1.88∼3.06)	<0.001	2.38 (1.87∼3.03)	<0.001	1.70 (1.32∼2.18)	<0.001	1.80 (1.39∼2.32)	<0.001
T3 (≥0.275)	5.46 (4.37∼6.83)	<0.001	5.35 (4.27∼6.69)	<0.001	3.10 (2.45∼3.92)	<0.001	2.75 (2.14∼3.54)	<0.001
*P* for trend		<0.001		<0.001		<0.001		<0.001

Data are presented as HRs and 95% CIs. Non-adjusted model: none. Model I: adjusted for age and gender. Model II: adjusted for Model II, plus CURB-65, PSI, COPD, and persistent lymphocytopenia. Model III: adjusted for Model III, plus intubation, ICU admission, mechanical ventilation, and vasoactive drugs. NPAR, neutrophil percentage-to-albumin ratio; HR, hazard ratio; CI, confidence interval; T, tertile; HR, hazard ratio; CI, confidence interval; Ref, reference; CURB-65, confusion, urea nitrogen, respiratory rate, blood pressure, age ≥ 65 years; PSI, pneumonia severity index; COPD, chronic obstructive pulmonary disease; ICU, intensive care unit.

### 3.4 Restricted cubic spline analysis

Furthermore, adjusting for confounders according to the Cox regression model III, restricted cubic spline analysis confirmed a linear association between NPAR and mortality (Supplementary Figure) (*P* for non-linearity > 0.05). The risk of mortality exhibited an upward tend with the rise in NPAR levels.

### 3.5 Subgroup analysis

**FIGURE 3:**
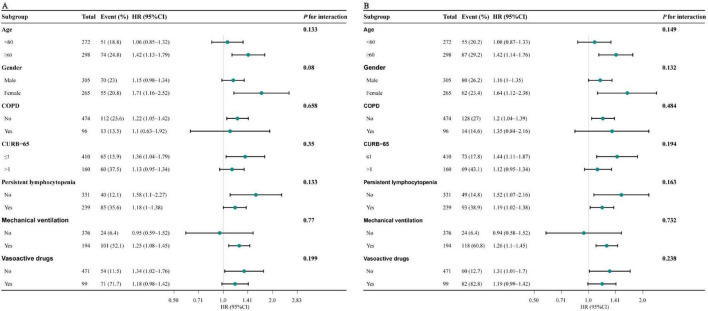
Forest plot for subgroup analysis of the association between NPAR and 30-day **(A)** and 90-day mortality **(B)**. Models adjusted for age, sex, CURB-65, PSI, COPD, persistent lymphocytopenia, intubation, ICU admission, mechanical ventilation, and vasoactive drugs. NPAR, neutrophil percentage-to-albumin ratio; HR, hazard ratio; CI, confidence interval; COPD, chronic obstructive pulmonary disease; CURB-65, confusion, urea nitrogen, respiratory rate, blood pressure, age ≥ 65 years; PSI, pneumonia severity index; COPD, chronic obstructive pulmonary disease; ICU, intensive care unit.

To further verify the robustness of the results, subgroup analyses were conducted according to the following stratification variables: age, gender, COPD, CURB-65, persistent lymphocytopenia, mechanical ventilation, vasoactive drugs. The trend of the effect size was consistent in all subgroups (Figures 3A, B). No interactions were identified within all subgroups (all *P* for interaction > 0.05).

### 3.6 Sensitivity analysis

To verify the stability of the results, we performed sensitivity analysis. After exclusion of 133 patients with chronic renal failure, liver failure, cirrhosis, nephrotic syndrome, congestive heart disease, or tumor at the baseline, the sensitivity analysis confirmed our findings that the NPAR was significantly associated with 30-day and 90-day mortality among patients with community-acquired pneumonia receiving systemic glucocorticoids therapy (Supplementary Table 2).

## 4 Discussion

In this retrospective cohort study, we revealed the association between higher NPAR levels and an increased risk of 30-day and 90-day mortality in patients who developed community-acquired pneumonia after receiving glucocorticoids therapy. The restricted cubic spline curve visually demonstrates this. Kaplan-Meier survival curves indicated that a higher NPAR was significantly associated with an increased risk of 30-day and 90-day mortality compared to lower NPAR values. Subgroup analysis found no significant interaction across different subgroups. The finding suggest that NPAR could serve as a potential biomarker for predicting mortality risk in clinical practice.

Prolonged use of systemic glucocorticoids at high doses may lead to lymphocytopenia and inhibit macrophage function in patients (25), thereby resulting in severe immunosuppression and consequently increasing the incidence of opportunistic infections, including CAP (7, 8, 10, 26). The study indicated that the incidence of CAP was notably increased in individuals receiving systemic glucocorticoids (equivalent to a daily dose of >20 mg prednisone), and was approximately threefold higher than in those not using systemic glucocorticoids. Agustí C et al. discovered that the overall mortality rate among patients undergoing on long-term oral glucocorticoids treatment was as high as 45% upon developing pulmonary infection, rising to 93% if mechanical ventilation was required (10).

Inflammatory responses play a pivotal influence in the development and progression of CAP (27). The neutrophil percentage, a crucial indicator of the body’s inflammatory response, is often significantly increased in pneumonia patients as a result of the immune system’s activation to counteract pathogens (28, 29). However, in patients receiving long-term glucocorticoids, this response is frequently dysregulated (30). Although neutrophil percentage is relatively high, their ability to eliminate pathogens is impaired due to the immune-suppressive effects of glucocorticoids (31). This “ineffective inflammation” can result in persistent pulmonary infections, thereby increasing the risk of severe complications and death. Albumin, a crucial protein with the body, has been employed as a biochemical marker for nutritional status, immune function, and systemic inflammatory state (29, 32). Inflammation leads to a decrease in serum albumin levels by suppressing hepatic albumin synthesis, increasing the production of inflammatory factors and impairing the vascular endothelium to prompt albumin leakage from blood vessels (33). Consequently, hypoalbuminemia is common in patients with CAP, especially those with severe CAP. Furthermore, in patients treated with long-term and systemic glucocorticoids, hypoalbuminemia may be exacerbated by several factors, including the promotion of protein catabolism by glucocorticoids (34), the reduction of hepatic albumin synthesis (35), and the increase in urinary protein excretion (36). Numerous studies have demonstrated that low albumin levels are correlated with mortality (37). The NPAR, a novel biomarker combining the neutrophil percentage and serum albumin, which is closely associated with the inflammatory response and nutritional status (38). In addition to long-term use of glucocorticoids, we found that the main reason for the decrease of albumin in the high NPAR group was an increased systemic inflammatory burden in the present study. Therefore, hypoalbuminemia, along with elevated NPAR, was significantly associated with increased mortality.

Several previous studies have demonstrated that NPAR was an effective marker for predicting mortality across populations. For instance, Maside Ari et al. reported that NPAR could potentially serve as a biomarker for predicting disease severity and mortality risk among pneumonia patients aged over 80 years (17). Another investigation has revealed that elevated NPAR levels were correlated with an augmented risk of 30-day and 90-day mortality among patients suffering from severe sepsis or septic shock (18). Similarly, in pneumonia patients with stroke (20) or traumatic spinal cord injury (39), NPAR has been demonstrated to be correlated with mortality. Consistent with prior findings, the present study also revealed that a higher NPAR was associated with an increased risk of mortality, particularly in the highest NPAR tertile, which had a significantly higher risk for 30-day mortality (HR: 3.19, 95% CI: 2.42–4.2) and 90-day mortality (HR: 2.75, 95% CI: 2.14–3.54). Additionally, a linear relationship was observed between NPAR levels and both 30-day and 90-day mortality.

Given the restricted predictive ability of inflammatory markers including leukocytosis, CRP, and PCT for mortality (15), certain studies have explored other measurable laboratory markers associated with prognosis in CAP patients treated with prolonged glucocorticoids. A study conducted by Xia et al. (40) revealed that the BUN/ALB ratio served as a prognostic indicator for unfavorable 30-day outcomes in these patients. Similarly, Bai et al. (41) demonstrated that an elevated level of the systemic immune inflammatory Index (SII) was an independent predictor for 90-day mortality in ILD patients complicated with pneumonia. Furthermore, Cheng et al. (42) proposed that LDH served as a prognostic indicator for 90-day mortality in CTD patients with pneumonia. In this study, we focused on a relatively high-proportion cohort of patients with COPD, ILD, bronchial asthma, CTD, IIP, hematological diseases, nephrotic syndrome, chronic glomerulonephritis, or other indications for glucocorticoids therapy. Furthermore, the association between NPAR and inflammation was more direct, rendering it more valuable for comprehensive evaluation in patients with CAP after receiving glucocorticoids therapy. Simultaneously, a notable correlation was identified between the NPAR and 30-day as well as 90-day mortality in this study population, even subsequent to the adjustment for multiple confounding factors. To our knowledge, there are currently no other studies on the association between NPAR and the prognosis of patients who developed community-acquired pneumonia after receiving glucocorticoids therapy. Our study, which focuses on this particular population, addresses a gap in the existing literature. Unlike complex scoring systems for assessing CAP severity, such as CURB-65 and PSI, which necessitate multiple clinical and laboratory parameters, NPAR consists of the neutrophils percentage and serum albumin, routinely measured in blood tests. For patients with CAP, even in the absence of imaging examinations, NPAR could still function as an effective marker for rapid risk assessments. Recent research indicates that NPAR may not only serve as a prognostic marker but also act as a potential indicator for guiding therapeutic strategies (43). Consequently, the early detection of elevated levels of the NPAR in CAP patients after receiving glucocorticoids treatment can help identify high-risk patients and assist clinicians in making precise decisions to improve outcomes.

The present study inevitably had several limitations. First, this study was a retrospective cohort study which might have introduced some bias. Second, NPAR was calculated from data obtained from the first blood tests upon admission. Consequently, dynamic observation of NPAR was not feasible. Random errors caused by using only the first blood result might be unavoidable. Third, due to the limitations in the original dataset, we were unable to collect detailed information about the patients’ dietary habits and did not analyze the effects of dietary habits on the association of NPAR with mortality. Fourth, our study lacked data regarding the prevalence of pneumonic infiltrations, which significantly affected the assessment of disease’s clinical progression and prognosis. Fifth, although we have made efforts to control possible confounders by using multivariate models, there still existed confounding factors that might potentially influence the results. Nevertheless, the findings require validation through large-scale prospective studies.

## 5 Conclusion

The present study revealed that an elevated NPAR was significantly associated with 30-day and 90-day mortality in patients who developed CAP during treatment with oral or intravenous glucocorticoids. NPAR is expected to serve as an important independent predictor for this specific population.

## Data Availability

The datasets presented in this study can be found in online repositories. The names of the repository/repositories and accession number(s) can be found in the article/Supplementary material.

## Footnotes

1
www.datadryad.org

2
https://www.r-project.org/
