# Supplementary material for: Association between neutrophil percentage-to-albumin ratio and mortality in patients with community acquired pneumonia receiving systemic glucocorticoids: a retrospective cohort study
Source: Front Med (Lausanne). 2025 Oct 13;12:1689323. doi: 10.3389/fmed.2025.1689323 (PMC12558152; doi:10.3389/fmed.2025.1689323)
Supplement: Supplementary file 1 [file Table_1.docx]

***Supplementary Material***

**Supplementary table1** Results of univariate cox regression analysis of 30-day and 90-day mortaltiy.

| **Variables** | **30d-mortality** | | **90d-mortality** | |
| --- | --- | --- | --- | --- |
|  | **HR(95%CI)** | ***P-value*** | **HR(95%CI)** | ***P-value*** |
| Age≥60 years, n (%) | 1.35 (1.15,1.59) | < 0.001 | 1.49 (1.28,1.74) | < 0.001 |
| Female, n (%) | 0.91 (0.77,1.06) | 0.219 | 0.89 (0.77,1.03) | 0.124 |
| COPD | 0.54 (0.42,0.7) | < 0.001 | 0.5 (0.39,0.64) | < 0.001 |
| ILD | 1.21 (1.04,1.42) | 0.015 | 1.39 (1.2,1.61) | < 0.001 |
| Asthma | 0.58 (0.31,1.08) | 0.083 | 0.5 (0.27,0.93) | 0.03 |
| CTD | 1.24 (1.06,1.45) | 0.008 | 1.29 (1.11,1.5) | < 0.001 |
| IIP | 0.89 (0.69,1.15) | 0.385 | 0.98 (0.77,1.23) | 0.851 |
| Nephrotic syndrome or CRF | 0.92 (0.73,1.15) | 0.453 | 0.95 (0.77,1.16) | 0.594 |
| CURB-65＞1 | 2.84 (2.43,3.32) | < 0.001 | 2.97 (2.56,3.44) | < 0.001 |
| PSI | 1.02 (1.01,1.02) | < 0.001 | 1.02 (1.02,1.02) | < 0.001 |
| White blood cell(×10^9^/L) | 1.04 (1.03,1.05) | < 0.001 | 1.04 (1.04,1.05) | < 0.001 |
| Neutrophil(×10^9^/L) | 1.07 (1.06,1.08) | < 0.001 | 1.07 (1.06,1.08) | < 0.001 |
| Lymphocyte(×109/L) | 0.62 (0.54,0.7) | < 0.001 | 0.63 (0.56,0.72) | < 0.001 |
| Hemoglobin(g/L) | 0.9925(0.9893,0.9957) | < 0.001 | 0.9918(0.9889,0.9948) | < 0.001 |
| Platelet(×109/L) | 0.9947(0.9937,0.9957) | < 0.001 | 0.995 (0.9941,0.9959) | < 0.001 |
| Albumin(g/dL) | 0.43 (0.38,0.49) | < 0.001 | 0.47 (0.42,0.53) | < 0.001 |
| AST(U/L) | 1.0018(1.0012,1.0025) | < 0.001 | 1.0018 (1.0012,1.0025) | < 0.001 |
| ALT(U/L) | 1.0034(1.0017,1.0051) | < 0.001 | 1.0032 (1.0015,1.0049) | < 0.001 |
| BUN (mmol/L) | 1.05 (1.04,1.05) | < 0.001 | 1.05 (1.04,1.06) | < 0.001 |
| Serum creatinine (mmol/L) | 1.0016 (1.001,1.0022) | < 0.001 | 1.0013(1.0007,1.0019) | < 0.001 |
| LDH(U/L) | 1.0009 (1.0007,1.001) | < 0.001 | 1.0009 (1.0008,1.001) | < 0.001 |
| ESR(mm/h) | 1.0038(1.0015,1.0061) | 0.001 | 1.0063(1.0042,1.0085) | < 0.001 |
| Procalcitonin (ng/mL) | 1.0076(1.0044,1.0107) | < 0.001 | 1.0077(1.0046,1.0108) | < 0.001 |
| NPAR(z-score) (dL/g) | 1.47 (1.4~1.54) | <0.001 | 1.46 (1.4~1.52) | <0.001 |
| Persistent lymphocytopenia | 3.38 (2.86,4) | < 0.001 | 3.09 (2.65,3.61) | < 0.001 |
| Oxygenation index | 0.9906(0.9897,0.9914) | < 0.001 | 0.9901 (0.9893,0.9909) | < 0.001 |
| Total pathogenic positive rate | 1.39 (1.07,1.8) | 0.015 | 1.48 (1.16,1.88) | 0.002 |
| Cumulative methylprednisolone dosages (g) | 0.99 (0.98,1) | 0.004 | 0.99 (0.98,0.99) | < 0.001 |
| Oxygeninhalation | 3.1 (2.44,3.94) | < 0.001 | 3.7 (2.92,4.7) | < 0.001 |
| Mechanical ventilation | 11.03 (9.04,13.47) | < 0.001 | 13.77 (11.31,16.77) | < 0.001 |
| Intubation | 5.04 (4.28,5.95) | < 0.001 | 6.18 (5.27,7.24) | < 0.001 |
| ICU admission | 8.15 (6.68,9.95) | < 0.001 | 9.99 (8.21,12.16) | < 0.001 |
| ECMO | 2.34 (1.81,3.03) | < 0.001 | 2.66 (2.11,3.36) | < 0.001 |
| Vasoactive drugs | 9.86 (8.4,11.58) | < 0.001 | 11.64 (10,13.56) | < 0.001 |
| CVVH | 5.84 (4.89,6.96) | < 0.001 | 6.13 (5.19,7.24) | < 0.001 |

**Note:** Data are presented as HRs and 95% CIs.

**Abbreviations: HR**, hazard ratio; **CI**, confidence interval; **COPD**, chronic obstructive pulmonary disease; **ILD**, interstitial lung diseases; **CTD**, connective tissue disease; **IIP**, idiopathic interstitial pneumonia; **Hematonosis**: anemia, leukemia, lymphoma, bonemarrow transplantation; **CURB-65**:confusion, urea nitrogen, respiratory rate, blood pressure, age≥65 years; **PSI**, pneumonia severity index; **LDH**, Lactate dehydrogenase; **BUN**, blood urea nitrogen; ****ESR****, **erythrocyte sedimentation rate**; **ICU**, intensive care unit; **ECMO**, extracorporeal membrane oxygenation; **CVVH**, continuous venovenous hemofiltration; **NPAR**, neutrophil percentage-to-albumin ratio.


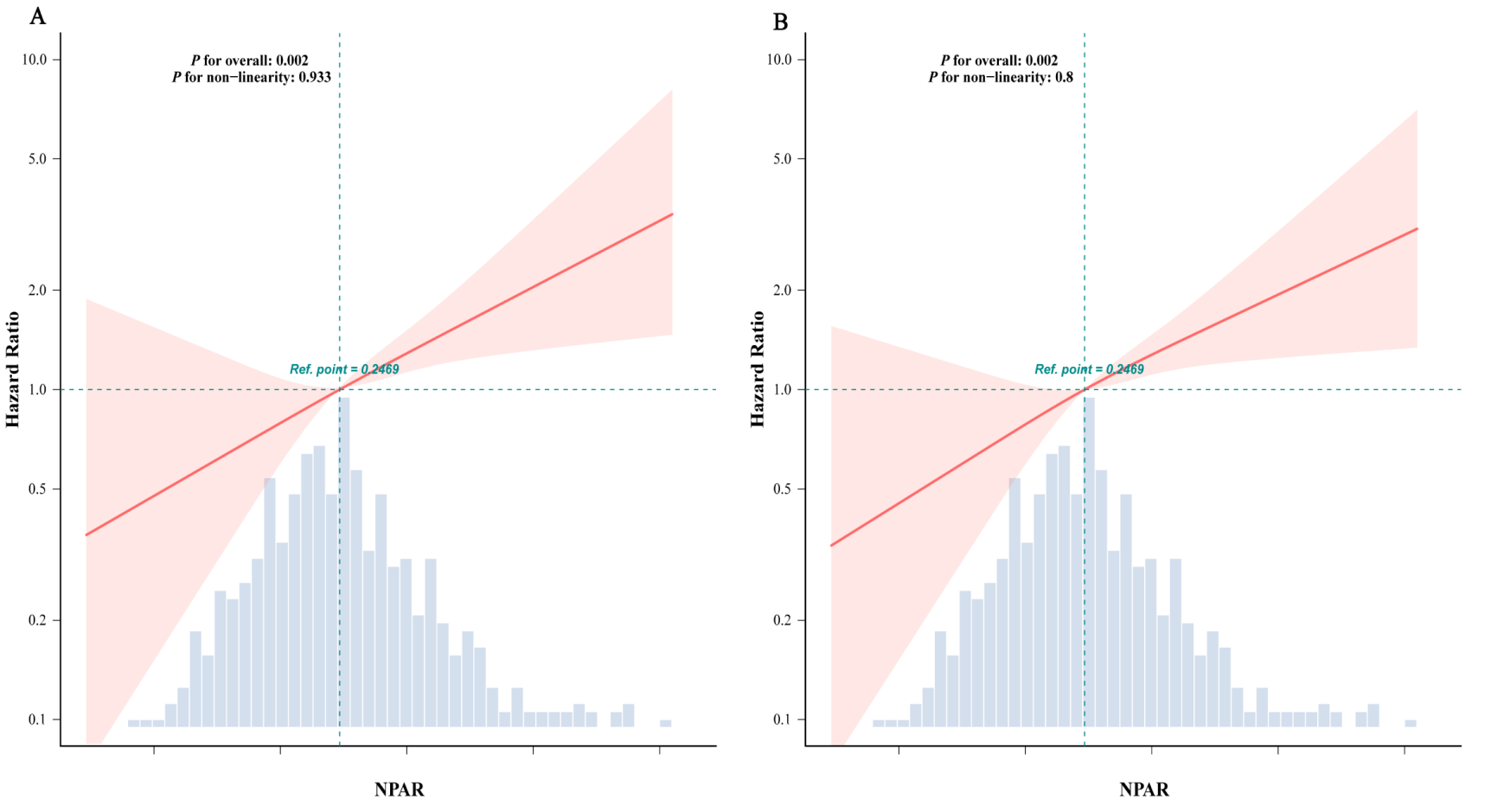


**Supplementary figure.** Association of neutrophil percentage-to-albumin ratio (NPAR) with 30-day(A) and 90-day mortality(B)(the highest and lowest 0.5% was trimmed for each NPAR measure).Adjusted confounders included age, gender, CUB-65, PSI, COPD, persistent lymphocytopenia, intubation, ICU admission, mechanical ventilation, and the use of vasoactive drugs.

**Abbreviations: NPAR**, neutrophil percentage-to-albumin ratio; **CURB-65**: confusion, urea nitrogen, respiratory rate, blood pressure, age≥65 years; **PSI**, pneumonia severity index; **COPD**, chronic obstructive pulmonary disease; **ICU**, intensive care unit.

**Supplementary table2.** Sensitivity analysis of the NPAR on 30-day and 90-day mortality in patients without chronic renal failure, liver failure, cirrhosis, nephrotic syndrome, congestive heart disease, or tumor (n=437).

| **Variable** | **Non-adjusted Model** | |  | **Model Ⅰ** | |  | **Model Ⅱ** | |  | **Model Ⅲ** | |
| --- | --- | --- | --- | --- | --- | --- | --- | --- | --- | --- | --- |
|  | **HR (95%CI)** | ***P*-value** |  | **HR (95%CI)** | ***P*-value** |  | **HR (95%CI)** | ***P*-value** |  | **HR (95%CI)** | ***P*-value** |
| **30-day mortality** | | | | | | | | | | | |
| **NPAR(Per SD increment)** | 1.93 (1.77~2.1) | <0.001 |  | 1.92 (1.77~2.1) | <0.001 |  | 1.6 (1.43~1.77) | <0.001 |  | 1.49(1.33~1.66) | <0.001 |
| **NPAR tertile** |  | | | | | | | | | | |
| T1（<0.218） | 1(Ref) |  |  | 1(Ref) |  |  | 1(Ref) |  |  | 1(Ref) |  |
| T2（≥0.218, <0.275） | 2.8 (2.09~3.75) | <0.001 |  | 2.77 (2.07~3.72) | <0.001 |  | 1.8 (1.33~2.44) | <0.001 |  | 2.14 (1.57~2.9) | <0.001 |
| T3（≥0.275） | 6.85(5.22~8.98) | <0.001 |  | 6.76 (5.15~8.87) | <0.001 |  | 3.53(2.63~4.73) | <0.001 |  | 3.44(2.53~4.68) | <0.001 |
| ***P* for trend** |  | <0.001 |  |  | <0.001 |  |  | <0.001 |  |  | <0.001 |
| **90-day mortality** | | | | | | | | | | | |
| **NPAR(Per SD increment)** | 1.9 (1.75~2.05) | <0.001 |  | 1.89(1.74~2.04) | <0.001 |  | 1.58 (1.43~1.75) | <0.001 |  | 1.45(1.31~1.61) | <0.001 |
| **NPAR tertile** |  | | | | | | | | | | |
| T1（<0.218） | 1(Ref) |  |  | 1(Ref) |  |  | 1(Ref) |  |  | 1(Ref) |  |
| T2（≥0.218, <0.275） | 2.69(2.06~3.51) | <0.001 |  | 2.64(2.02~3.45) | <0.001 |  | 1.81 (1.37~2.38) | <0.001 |  | 2.14(1.62~2.83) | <0.001 |
| T3（≥0.275） | 6.12(4.78~7.85) | <0.001 |  | 5.97(4.65~7.65) | <0.001 |  | 3.18 (2.43~4.17) | <0.001 |  | 3.02(2.27~4.01) | <0.001 |
| ***P* for trend** |  | <0.001 |  |  | <0.001 |  |  | <0.001 |  |  | <0.001 |

Data are presented as HRs and 95%Cls.

Non-adjusted model: none.

Model I: adjusted for age and gender.

Model II: adjusted for Model II, plus CURB-65, PSI, COPD, and persistent lymphocytopenia.

Model III: Adjusted for Model III, plus intubation, ICU admission, mechanical ventilation, and vasoactive drugs.

**Abbreviations:** NPAR, neutrophil percentage-to-albumin ratio; HR, hazard ratio; CI, confidence interval; T, Tertile; HR, hazard ratio; CI, confidence interval; Ref, reference; CURB-65: confusion, urea nitrogen, respiratory rate, blood pressure, age≥65 years; PSI, pneumonia severity index; COPD, chronic obstructive pulmonary disease; ICU, intensive care unit.
